# Reconsidering the role of the IL-23/IL-17 immune axis in idiopathic nephrotic syndrome pathogenesis

**DOI:** 10.1093/ckj/sfad264

**Published:** 2023-10-13

**Authors:** Giuseppe Salfi

**Affiliations:** Oncology Institute of Southern Switzerland, Bellinzona, Switzerland

To the Editor,

We read with great interest the manuscript authored by Stryckers *et al.* [1] that presents a unique case report highlighting drug-induced focal segmental glomerulosclerosis (FSGS) in a patient undergoing interleukin-23 (IL-23) inhibitor treatment with guselkumab. This work not only unveils a novel secondary mechanism of FSGS development, but also provides stimulating insights that shed light on an important proposed element in the pathogenesis of the idiopathic forms of nephrotic syndrome (INS) and FSGS: the IL-23/IL-17 immune axis.

The pathogenesis of INS has long perplexed the medical community, with numerous molecular mechanisms identified but often remaining disjointed and challenging to unify into a comprehensive framework. A well-known hypothesis involves impaired activation of T-regulatory (Treg) cells and concurrent upregulated T-helper 17 (Th17) cell function, which is thought to damage the glomerular filtration barrier [2].

*In vitro* studies have demonstrated that both Th17 cell supernatants from healthy donors and plasma from NS patients can boost podocyte motility through the c-Jun N-terminal kinase (JNK) pathways [3]. Clinical investigations in adult minimal change disease patients have linked a higher Th17:Treg ratio with increased proteinuria and decreased albumin levels, with a subsequent reduction in this ratio following successful treatment [4].

Further studies showed increased expression of IL-17 in kidney biopsies of paediatric INS patients and higher Th17 cell counts in their blood samples compared with controls [5, 6]. Higher circulating levels of Th17-related cytokines (IL-23 and IL-17) have been found in the plasma of INS patients compared with healthy controls [4] and in relapsing INS patients compared with those in remission [7].

Finally, in 2018, Zhang *et al.* [8] proposed a pathogenetic model of INS in which γδ T cells were stimulated by IL-23 to secrete IL-21, ultimately leading to the imbalance in Th17/Treg cells.

Moving back to the case report by Stryckers *et al.* [1], the rapid remission of NS upon discontinuation of guselkumab treatment strongly suggests a plausible causal link between IL-23 inhibitor therapy and FSGS development. Similarly, a 2019 case report documented the emergence of FSGS and NS in a patient treated with the anti-IL-12/IL-23 antibody ustekinumab, which subsequently resolved upon treatment cessation [9].

In light of these recent findings, it becomes imperative to contemplate whether it is conceivable for both the activation and inhibition of the IL-23/IL-17 axis to lead to FSGS and INS. Some could argue that INS may represent a diverse group of diseases with distinct pathogenic mechanisms, making it difficult to generalize findings from one subgroup to another. Furthermore, the absence of FSGS development in the patient subsequently treated with IL-17 inhibitor secukinumab could imply that this side effect may be specific to guselkumab rather than the overall inhibition of the IL-23/IL-17 axis.

However, in conclusion, it should not be forgotten that the involvement of the IL-23/IL-17 axis in the pathogenesis of INS is still a disputed matter, lacking solid experimental and clinical support, and therefore remains yet to be proven.
